# Stakeholder identified research priorities for early intervention in psychosis

**DOI:** 10.1111/hex.13604

**Published:** 2022-09-21

**Authors:** Laoise Renwick, Caitlin McWilliams, Olivia Schaff, Laura Russell, Susan Ramsdale, Rebecca Lauren Morris

**Affiliations:** ^1^ School of Health Sciences, Division of Nursing, Midwifery and Social Work, Faculty of Medicine, Biology and Health University of Manchester Manchester UK; ^2^ The Education Campus ‐ Oxford Road Central Manchester Foundation NHS Trust Library Services Manchester UK; ^3^ The Education and Research Centre – Wythenshawe Hospital Central Manchester Foundation NHS Trust Manchester UK; ^4^ NIHR Greater Manchester Patient Safety Translation Research Centre,Division of Population Health, Health Services Research and Primary Care School of Health Sciences Manchester UK

**Keywords:** early intervention in psychosis, early psychosis, research priority, user participation

## Abstract

**Background:**

Public resources to answer pertinent research questions about the impact of illness and treatment on people with mental health problems are limited. To target funds effectively and efficiently and maximize the health benefits to populations, prioritizing research areas is needed. Research agendas are generally driven by researcher and funder priorities, however, there is growing recognition of the need to include user‐defined research priorities to make research more relevant, needs‐based and efficient.

**Objective:**

To gain consensus on top priorities for research into early intervention in psychosis through a robust, democratic process for prioritization enlisting the views of key stakeholders including users, carers and healthcare professionals. We also sought to determine which user‐prioritized questions were supported by scientific evidence.

**Design and Methods:**

We used a modified nominal group technique to gain consensus on unanswered questions that were obtained by survey and ranked at successive stages by a steering group comprising users, carer representatives and clinicians from relevant disciplines and stakeholder bodies. We checked each question posed in the survey was unanswered in research by reviewing evidence in five databases (Medline, Cinahl, PsychInfo, EMBASE and Cochrane Database).

**Results:**

Two hundred and eighty‐three questions were submitted by 207 people. After checking for relevance, reframing and examining for duplicates, 258 questions remained. We gained consensus on 10 priority questions; these largely represented themes around access and engagement, information needs before and after treatment acceptance, and the influence of service‐user (SU) priorities and beliefs on treatment choices and effectiveness. A recovery SUtheme identified specific self‐management questions and more globally, a need to fully identify factors that impact recovery.

**Discussion and Conclusions:**

Published research findings indicated that the priorities of service users, carers and healthcare professionals were aligned with researchers' and funders' priorities in some areas and misaligned in others providing vital opportunities to develop research agendas that more closely reflect users' needs.

**Patient and Public Contribution:**

Initial results were presented at stakeholder workshops which included service‐users, carers, health professionals and researchers during a consensus workshop to prioritize research questions and allow the opportunity for feedback. Patient and public representatives formed part of the steering group and were consulted regularly during the research process.

## 1 INTRODUCTION

Limited resources place constraints on healthcare research and necessitate prioritization of scarce funds to effectively select research with maximum health benefits for populations. Research agendas are typically agreed upon at strategic levels by government officials and experts. Few strategic research plans exemplify community participation and the criteria and mechanisms that guide how decisions are made lack transparency.^1^ Comprehensively, reviewed literature shows that when patients and clinicians are invited to contribute their opinions and views about their priorities for research, they are disregarded in determining subsequent agendas and policies.^2^ Importantly, another review found that strategic decisions are frequently underpinned by estimates of the magnitude of disease burden and the desire for social justice.^3^ However, other factors identified may contribute to inequity in setting research agendas including prioritizing potential high‐impact publications, matching research goals with capacity, prioritizing research interests of academics and financial and political interests of donors.^3^, ^4^ Research agendas influenced by political and social drivers other than disease burden and social need can create gaps in knowledge that influence the availability of evidence‐based treatment and can under‐prioritize research on some illnesses compared to burden.^1^ Moreover, providing mental healthcare based on uncertain evidence can potentially compromise patient care and wastes valuable resources.^5^

Uncertainty can influence patient outcomes across all areas of decision‐making in mental health including assessing intake acceptance, assigning diagnoses, attributing causal explanations and treatment and care planning.^6^ In psychotic illness, variation in recognition, assessment and intervention has led to delays in treatment receipt and delivery.^7^ Despite considerable advances in research, some prognostic and treatment uncertainty remains.^8^ Evidence shows that the treatment gap is high, particularly among young people. Less than a third of young people do not seek professional mental healthcare^9^ and there are high rates of unmet need as evidenced by variable invitation and uptake rates of NICE‐approved therapies for serious mental illness.^7^, ^10^ Despite this, there is a good return on investment in research on mental health. Comprehensive economic analysis of evidence‐based treatments for mental health problems shows that funding spent on mental health research yields observable benefits in direct patient care and wider socioeconomic gains.^11^

Researchers are beginning to acknowledge that scientifically important questions may not be directly relevant to patients^12^ which is believed to be a contributing factor to knowledge translation gaps between healthcare delivery and the application of evidence.^13^ Increasing the availability of user‐prioritized, high‐quality research evidence has considerable potential, among other methods, to reduce avoidable waste in healthcare research,^5^ accelerate knowledge translation,^14^, ^15^ ensure research is acceptable and appropriate to consumers^15^ and make researchers more accountable for public expenditure, ensuring research that is relevant and applicable in the real world.^16^ Actively involving users (clinicians, service users and carers) is now a key criterion for commissioning research from the National Institute for Health Research (NIHR).^17^ The objective is to enhance research processes and outputs by an authentic partnership between consumers and researchers sharing responsibility for planning, conduct, interpretation, dissemination and adoption of research findings.^18^

The aim of this project was to generate a set of user‐identified research priorities for individuals in the early phase of psychosis. This is a fertile area of research. The development of specialist research units, regional, pan‐continental, and international networks, field‐specific conferences, journals and citation indices evidence this. Some researchers argue that bringing research interest and priorities closer to the need and demand for services is essential to identify and prioritize gaps in knowledge,^19^ therefore, we aimed to develop priorities that were currently unanswered by research for early psychosis care and treatment.

## 2 METHODS

Using mixed methods, we systematically identified and prioritized unanswered questions in early psychosis research. We implemented a staged process of surveying to gather priorities, systematic literature searches to determine unanswered questions and a modified nominal group workshop to determine priorities. Our methods were informed by the recommendations of Chalmers et al.^5^ and Nasser et al.^20^; to engage stakeholders and the wider early intervention (EI) community meaningfully, draw on existing priorities and reviews, ensure the team was knowledge‐ready, and pilot, assess and revise processes periodically. As we aimed to understand the needs, preferences and priorities for research about early psychosis from the perspectives of users of research rather than users of healthcare services we adopted a multidisciplinary approach to involve a broad representation of stakeholders.^21^ Good practice principles for priority‐setting exercises promote inclusion of multisectorial and multidisciplinary stakeholders to ensure different perspectives are considered, a broad range of relevant priorities can be obtained and to establish a network to foster ownership among stakeholders to encourage fair representation among groups^22^ and we used these principles in generating priorities for research users.

### 2.1 Steering group initiation and survey development

The steering group (VIP‐SG) was established to shape the survey question and design the survey, determine the scope of the project and comment on the processes and procedures for identifying, ranking, and prioritizing uncertainties, agreeing on methods and providing expertise in gaining final consensus. The VIP‐SG comprised service user representatives, clinical representatives, information specialists, leadership and administrative support. Representatives were selected for their involvement with relevant professional groups to represent multiple professional disciplines, user–researcher involvement, and user and carer representation.^21^, ^22^ The VIP‐SG met on three occasions during the project and was consulted periodically about decisions at key points. A full list of members is provided here (http://www.vip.bmh.manchester.ac.uk/).

We identified key themes among relevant policies, guidelines and performance targets as a starting point for discussions around the scope of the project, initially asking the group to consider the appropriateness of the themes and generate further discussion. These included the Mental Health Programme Implementation Guide 2001,^23^ National Institute for Health and Care Excellence (NICE) Guidelines for Early Psychosis,^24^ Commissioning for Quality and Innovation National Goals (CQUINS) 2017/18, Achieving Better Access to Mental Health Services by 2020/21,^25^ the Early Psychosis Declaration 2005^26^ and the Five Year Forward View 2016.^27^

### 2.2 Gathering responses

Responses to questions were gathered during two phases of the survey and these were delivered online on both occasions. We piloted the survey in the first phase to identify any implementation issues and ensure high‐quality data. The Phase 1 survey was developed by the VIP‐SG adopting a comprehensive approach to generating uncertainties reflecting the wide‐ranging research areas within EI. These included all aspects of illness, recovery, and treatment given that psychotic illnesses are complex, and outcomes of psychosis and subthreshold illnesses framed in a transdiagnostic framework are heterogeneous. Questions were developed in six themes covering community engagement, recovery, treatment refractoriness, effective interventions, EI and physical health improvement (see Supporting Information for Survey 1). We reviewed processes with the steering group and utilized specific feedback from service‐user (SU) representatives due to a high number of partial responses within the pilot data. Phase 2 survey was then refined to simplify the question format into a single question regarding research priorities rather than utilizing a thematic approach (see Supporting Information for Survey 2). Intensive efforts were taken during Phase 2 to publicise the survey and increase participation. The VIP‐SG promoted the online surveys among their networks, and we used social media to attract attention and encourage completion targeting a broad range of disciplines including nurses, psychiatrists, occupational therapists, psychologists, general practitioners, social workers and police officers. During Phase 2, the first author reviewed NHS Trust websites for user involvement group information and where available, contacted user involvement leads to publicise within their organizations and make the survey available in EI networks online and mental health user groups and charities.

### 2.3 Indicative question development

Once the final survey closed, data were merged, and questions were reviewed by two team members (L. R. and R. L. M.). The first author reviewed submissions twice, initially developing indicative questions and on the second review developing and assigning each question to a theme and subtheme which were used to organize the data and identify duplicates. Indicative questions were developed where there were multiple questions with similar meanings and these were assigned red, amber and green classifications based on the degree of confidence held by the researcher regarding how accurately the question retained its intended meaning based on the participants' submission. A consensus meeting between L. R. and R. L. M. was held to determine iterative questions and questions, not in remit which was removed, for example, questions out of scope, philosophical questions not answerable by research and basic research. Iterative questions are determined following an iterative process of refining the questions and the purpose was twofold; we aimed to refine questions to ensure they were researchable and answerable and to ensure that we retained the meaning of each question posed, whether framed in commentary or question, to the greatest extent possible. When respondents asked about NHS service access we considered an effectiveness question to precede this, that is, if a question asked about cognitive behavioural therapy (CBT) access we included a question about whether CBT was effective. Other examples include home treatment, routine physical health screening, public and targeted education, self‐management and supported employment. The iterative process of refining and reformulating questions was based on the agreement between two researchers (L. R. and R. L. M.). See Table 1 for examples of original submissions, and indicative and iterative questions.

**Table 1:** Iterative question formulation

Original question	Iterative question	Certainty (green/amber/red)
SU's experience of/preference for/and the outcomes of therapies other than CBT for early psychosis. BME SU's preferences for therapy with EI teams/factors that affect BME SU's engagement with therapy within EI. The impact of the EI standards on patient care/staff effectiveness/staff morale/overall service delivery.	What are service‐users preferences for psychological treatments during a first episode of psychosis?	Amber
	What is the effectiveness of different psychological interventions during a first episode of psychosis?	Green
	What are the treatment priorities for people of black and ethnic minority groups in early intervention services?	Green
	What factors influence engagement with treatment for people in black and ethnic minority groups?	Green
	What has been the impact of new waiting times standards on patient care, staff efficiency, morale and overall service delivery?	Green
Preamble: a Carer is utterly isolated when the Cared‐for suffers a psychotic episode, particularly when outside normal working hours and services aren't available. It's the police who come to the rescue very often. They have been very understanding. Often, staff at the [general]hospital see a mentally ill patient as a time waster. On one occasion, in A&E, my partner asked to see the on‐call psychiatrist but was refused until she'd had a breathalyser test [she hadn't been drinking]. She left. Question: why isn't there a uniform high standard of Care and empathy for the mentally ill?	What are the experiences of carers during acute episodes of psychosis outside normal working hours?	Red
How best to normalize experiences and maximize function using commercial and nonstigmatizing [sic] intervention	How can functioning be improved through the use of commercially available interventions?	Green
What could help the general public in advert style like the ones currently for mental health or stoke advise be produced that could help raise awareness and improve acceptance within communities and within schools	Does public and targeted education enhance awareness of psychosis?	Green
Why don't staff diagnose you so that you can research your illness?	What is the influence of delay in receiving a diagnosis on service‐users understanding of the illness?	Red
How do early symptoms [sic] impact on day to day activities? How long does it take an individual to ask for help?	What is the effect of psychotic symptoms on daily activities?	Amber

Abbreviation: BME, black and minority ethnic; CBT, cognitive behavioural therapy; EI, early intervention; SU, service‐user.

### 2.4 Search strategy

The search strategy was developed by an information specialist (O. S.) consulting with the wider specialist library team at Central Manchester Foundation NHS Trust and the first author (L. R.). For each question a systematic search of the literature was conducted in Medline, EMBASE, Cinahl, PsycINFO, Cochrane Database of Systematic Reviews and the Cochrane Controlled Register of Trials (CENTRAL) where applicable. Specific search strategies were developed for each database and for each question, using a combination of keywords and MeSH/Map terms to include the following: ‘psychosis’, ‘first‐episode psychosis’, ‘early intervention’ and question‐specific keywords. No limits were set on the publication date and level 1 evidence was retrieved comprising systematic reviews, meta‐analyses or qualitative metasyntheses (to yield returns relevant to qualitative questions that may include realist reviews, mixed methods or mixed studies reviews). We included reviews that focused on psychosis and schizophrenia spectrum diagnoses to extract information about first‐episode illnesses if available. Searches were conducted by a team of five information specialists at Manchester Royal Infirmary Library at Central Manchester Foundation Trust and Wythenshawe Academy Library at University Hospital of South Manchester NHS Foundation Trust between February and May 2018. In addition, representatives from VIP‐SG were asked to contribute papers they believed were relevant to answering the questions we obtained. L. R. checked the results and supplemented them with known systematic reviews that were not obtained during searches where available. The search results were analysed and evidence summaries were developed for each question where available and a question was considered to have met the certainty criterion if a systematic review concluded that there was sufficient evidence to answer the question.

### 2.5 Prioritization

All questions taken forward to prioritization were ranked in order of the 30 most important questions via an online survey by each VIP‐SG member and these were collated to develop a shortlist of priorities among treatment and illness uncertainties. VIP‐SG members, patients and carers were then recruited to participate in the final prioritization workshop using a modified nominal group technique. Attendees ordered the organization of questions where necessary, voted on their priorities among similarly themed questions and gained consensus on final priorities through small‐group discussions. The final 10 were agreed upon by the entire group during the consensus workshop which comprised service‐users, carers, healthcare professionals, advocates and researchers. Each member was allocated equal voting weight.

### 2.6 Ethical considerations

We adhered to ethical principles contained in organizational policies, but no identifying personal data were obtained and approval by University Research Ethics Committee was not required.

## 3 RESULTS

### 3.1 Question gathering and identifying iterative questions

Two hundred and seven people answered all or part of the free‐text surveys; there were 80 responses to the Phase 1 survey and 137 responses to the Phase 2 survey. See Table 2 for background for respondents who provided this data. Some respondents provided no question, commentary containing a question and some provided multiple questions; in total, the survey yielded 283 priority questions once submissions had been reviewed for iterative questions. Two hundred and fifty‐eight remained once out of scope and duplicate questions were removed. Thematic analysis and clustering into themes and subthemes determined 11 subthemes largely consistent with the classification developed for the preliminary stages of the project. In addition, subthemes comprised user‐applied basic research, crisis care, research funding and service models and delivery. The most prominent theme was ‘Promoting Recovery’ with almost half the questions posed relating to accessing effective interventions during recovery and predicating or enhancing recovery. Table 3 provides information about the thematic classification. Many questions related to promoting self‐management, peer support, understanding the impact of stigma and discrimination and questioning the influence of value‐based care.

**Table 2:** Service‐user, carer or healthcare professional status of participant

Participant	*n*	%
Person with psychosis	27	16
Partner/relative/friend of someone with psychosis	25	15
Nurse	22	13
Occupational therapist	47	28
Psychiatrist	10	6
Psychologist	11	7
Support worker	3	2
General practitioner	2	2
Social worker	3	2
Police officer	1	1
Other professional	7	4
Other	9	4
Total	165	

**Table 3:** Thematic classification

Theme	Frequency, *n* (%)
Promoting Recovery	123 (47.5)
Improving Access, Engagement and Treatment	36 (14)
Promoting Physical Health	23 (9)
Family Engagement and Support	18 (7)
Service Models and Delivery	16 (6)
Raising Community Awareness	15 (6)
Preventing Psychosis	9 (4)
User‐applied Basic Research	9 (4)
Practitioner Training	6 (2)
Crisis Care	2 (1)
Research Funding	1 (0.5)

### 3.2 Evidence searching and prioritization

It took 248 h of librarian time to conduct searches of the databases against the iterative questions comprising over 1000 searches. Relevant systematic reviews were returned in searches for 52 (18.4%) of the iterative questions and the number of available reviews for each question that had a review ranged from 1 to 10 reviews with varying degrees of evidence provided in each review. There were several areas where the evidence was extensive and robust which were also equivocal given the nature of the question posed, for example, no outcome identified, too broad to consider the findings to answer the question wholly or not first‐episode specific. These areas were the effect of lifestyle and diet interventions, the effect of public education campaigns on enhancing awareness, increasing help‐seeking and reducing the duration of untreated psychosis, the effect of family interventions, the effect of interventions to promote occupation, labour force participation, recreation and social network enhancement, prevention of psychosis, tailored interventions to manage weight gain, the effect of psychological interventions and cognitive remediation, influence of cannabis use and causal mechanisms in psychosis. There were reviews that gathered data on schizophrenia, but conclusions could not be drawn about the first episode or the early phase of illness, for example, managing sexual risk and safety, the effect of home‐based treatment and the long‐term effect of novel pharmacological treatments at this stage of illness. There were several qualitative metasyntheses exploring views about medication effectiveness, choice and lifestyle interventions, positive aspects of psychotic experiences, and the role of social support and networks during early psychosis. None of the systematic reviews reported a conclusive answer and we brought forward all questions for prioritization.

**Figure 1:**
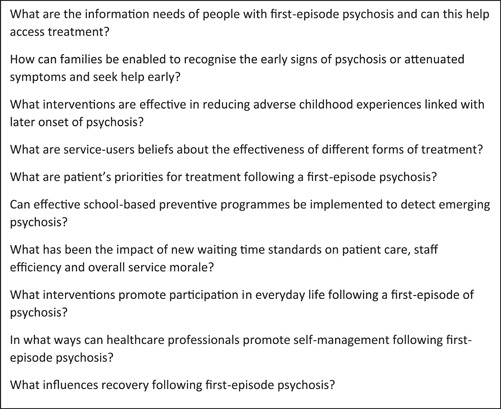
Top 10 user‐developed priorities for research in early intervention in psychosis

From 258 priority questions, there were 237 that were endorsed at least once by members of the steering group. Members of the group ranked their top 30 priority questions and once collated, we took forward 42 questions for final prioritization; included questions were endorsed four or more times. The final consensus workshop was attended by service‐users (*n* = 4), caregivers/family members (*n* = 4), clinicians/clinical leads (*n* = 8), policy administrators (*n* = 1) and educators (*n* = 2). See Table 4 for the shortlist of questions taken forward for final prioritization. Questions were arranged in their themes for the final voting and consensus was gained through anonymized voting for each priority question contained in the final list. For two questions, a consensus was obtained by group discussion and iterative voting to achieve consensus. Figure 1 contains the final priority list.

**Table 4:** Shortlist priority questions

Question	Votes	Theme
Is there a link between psychosis and social exclusion?	4	Basic Research
What is the availability of home treatment for people with first‐episode psychosis?	6	Crisis Care
What is the role of social networks in supporting people with psychosis?	4	Family Engagement and Support
What factors influence engagement with treatment for people in black and ethnic minority groups?	7	Improving Access, Engagement and Treatment
What are the information needs of people with first‐episode psychosis and can this help access treatment?	5	
How do people with psychosis interact with mental health services?	4	
What is the experience of the onset of psychosis?	4	
What interventions are effective in reducing adverse childhood experiences linked with later onset of psychosis?	4	Preventing Psychosis
What help can family's access within a primary care setting when the person with psychosis does not recognize they have an illness?	4	
How can families be enabled to recognize the early signs of psychosis or attenuated symptoms and seek help early?	4	
How can we support the sexual health of people with psychosis (i.e., managing sexual side effects of medication, managing impact of anxiety on sexual functioning, supporting the use to safe sexual practices in people who become ‘sexually disinhibited’)?, What predicts weight gain following antipsychotic medication initiation?	5	Promoting Physical Health
What is the role of psychiatrists in reducing the iatrogenic effects of psychiatric medication?	4	Promoting Physical Health
What interventions can help people with psychosis maintain their friendships and social networks?	6	Promoting Recovery
What are service‐users preferences for psychological treatments during a first episode of psychosis?	6	
Do people attending treatment in CMHT following EI experience similar rates of recovery compared to when attending EI services?	5	
What interventions are effective in retaining remission and recovery achieved in EIP when transferred to CMHT?	5	
How can treatments be more specific for different diagnostic categories of psychosis?, Can care coordinators effectively implement a full range of effective EIP treatments?	5	
Are community‐based resources effective in increasing recovery following first episode psychosis?	5	
In what ways can healthcare professionals promote self‐management following first‐episode psychosis?	5	
What is the impact of culture on understanding of psychosis?	5	
Can we identify the risk of treatment refractory illnesses at first presentation?	4	
Do the views of healthcare professionals towards psychosis outcome impact on the likelihood of recovery?	4	
What is the availability of employment support for people with experience of psychosis?	4	
What influences recovery following first‐episode psychosis?	4	
What do people with psychosis value about peer‐led interventions?	4	
What interventions promote participation in everyday life following a first episode of psychosis?	4	
In what way can people with psychosis be assisted to explore multiple meanings of psychotic symptoms/phenomena to support recovery?	4	
How do people who experience psychosis ascribe meaning to their experiences?	4	
Are people with psychosis from marginalized groups less likely to recover?	4	
What are patient's priorities for treatment following a first‐episode psychosis?	4	
How do people with psychosis maintain social networks in occupational roles?	4	
What are services‐users beliefs about the effectiveness of different forms of treatment?	4	
How is the terminology used to describe psychosis and outcome being perceived by service‐users?	4	
Can care coordinators effectively implement a full range of effective EIP treatments?	4	
Do public and targeted education campaigns reduce the stigma of psychosis?	5	Raising Community Awareness
Can effective school‐based preventive programmes be implemented to detect emerging psychosis?	4	
Comparative to other mental illnesses, do people with psychosis experience more discrimination?	4	
Do public and targeted education campaigns enhance awareness of psychosis, increase help‐seeking and reduce duration of untreated psychosis?	4	
What are the research funding decisions comparatively between long‐term physical conditions and long‐term mental health conditions?	6	Research Funding
What are the support needs of people with lived experience of psychosis to actively participate in roles around service delivery?	5	Service Models and Delivery
What has been the impact of new waiting time standards on patient care, staff efficiency, morale and overall service delivery?	5	
What is the experience of assessment, diagnosis and treatment in early intervention services versus CMHT?	4	

## 4 DISCUSSION

A final list of 10 priorities was obtained by consensus agreement by stakeholders comprising users of EI services and clinicians and can be considered an important record of user‐defined uncertainties providing intelligence concerning unanswered questions fundamental to the EI paradigm. Knowledge of uncertainties and unanswered questions is a necessary foundation for identifying future directions in research and can support international research efforts towards advancing early psychosis care and treatment. A key uncertainty involved preventing delays and promoting access to treatment and few reviews focused on this despite its centrality to the EI paradigm. Remarkably few studies examined the needs of users for information before gaining access to treatment or school‐based programmes to detect emerging psychosis. Meta‐analytic and narrative reviews emerged evaluating complex, multifocal interventions, discrete professional and patient levels initiatives and different service configurations for shortening delays in accessing care.^28^, ^29^

Prioritizing reducing delays aligns strongly with national research strategy and frameworks recommending a focus on prevention, earlier detection and treatment of mental illness^30^ citing current mental health policy.^27^ Promoting earlier access emerged strongly in several user‐defined priorities reflecting a need to more fully appreciate and systematise the intrinsic and extrinsic factors contributing to longer delays. The top 10 reflect uncertainties about the mechanism of action of interventions, user and carer needs for communicating information, the optimal development phase to target and the effectiveness of public health approaches. Fundamentally, the uncertain nature of the range of interventions targeting delay signals a need to systematically develop interventions to optimize mental health literacy and increase access to care. Specifically, there were no reviews exploring the barriers and facilitators of help‐seeking from the perspective of service users and carers, which is an important unanswered element of this complex puzzle.

The top 10 emphasized an absence of evidence for interventions to enhance community participation and increase users' ability to self‐manage. Reviews obtained in searches largely dealt with recovery using researcher‐defined outcomes.^31^, ^32^ User‐defined prioritized outcomes were largely absent and there was an imbalance with reviews weighted towards estimating rates and predictors of recovery. This is consistent with the UK healthcare research funding landscape where basic and user‐applied basic research dominates; most of the funding is allocated to research that will not immediately benefit users.^14^, ^33^ Fewer available reviews investigated active interventions for recovery including self‐management and social media applications,^34^ interventions to enhance social participation and networks,^35^, ^36^ peer support interventions^37^ and engagement in meaningful activities^38^ with none of the reviews solely addressing the needs of people during the early stages of psychosis at the time of analysis. As clinically applicable research which leads to larger health, social and economic gains than basic research^39^ efforts towards redressing this imbalance are essential.

Indeed, a prominent question considered by the consensus group asked specifically about how research funding decisions were made, this question was not retained in the final 10 but captures the essence of this project in that ultimately these priorities will be used by funders and researchers to identify future research priorities and carve out strategic pathways to conduct research of relevance to service users, carers and healthcare professionals. Prioritization is only of value if research follows^40^ which can justify the importance and influence of these types of collaboration,^40^ but it is crucial in future partnerships with funders to ensure priorities are refined and aligned with funders' strategies. In terms of the available evidence, there were some areas of strength where significant volumes of research evidence had amassed. Importantly for user involvement research, this signifies a degree of alignment between early psychosis research and service‐user, carer and healthcare professionals' views and priorities. These were management of weight gain, lifestyle and diet interventions, exercise interventions, effectiveness of family interventions, prevention of transition to psychosis and relapse, cannabis use and psychosis risk, causal factors for psychosis and digital health interventions. Some of these findings are considered particularly robust and have informed clinical guidelines and policy.^24^, ^25^

## 5 STRENGTHS AND LIMITATIONS

This project was a substantial group effort from the VIP‐SG to engage relevant stakeholders in identifying priorities. Targeted activities such as stakeholder review with collaborative networks for applied research, engagement and presentation to user involvement groups, service‐user research groups and charities, and a strong online and social media presence have been a successful strategy eliciting pertinent questions from interested groups. Many questions posed were specific to the field and while relevant, iterative questions tended towards questioning the effectiveness, value and availability of general mental healthcare and treatment.^41^ Arguably, lack of specificity might lessen the perceived importance of some of the identified priorities. This reflects the nature of collaboration and promoting meaningful representation of user views captures the ideology of user involvement in research prioritization. The findings are indicative of the views of stakeholders included though generalizability cannot be assumed as we did not obtain a random sample of individuals working in and attending early psychosis services. It is noteworthy, however, that research has shown that the stated priorities of self‐selecting groups of service users are similar to that of participants who are randomly selected to provide their views.^42^

To promote nontokenistic involvement, efforts need to be taken to curb power imbalance and ensure active involvement from service users in mixed stakeholder groups.^43^ We took measures to minimize imbalance and foster equal participation throughout phases of gathering and agreeing on uncertainties, such as anonymized surveys and secret voting on priorities. However, we met with difficulty in recruiting individuals that were able to commit to longer‐term involvement such as steering committee membership. As per NIHR guidance, service‐user involvement in the steering group was conducted separately from usual committee business to encourage authentic participation, however, it may have been more beneficial to set up an additional lay advisory group to focus solely on service‐user and carer input to the research.^44^

Lastly, methodologies used to obtain evidence to answer user‐defined questions were predominantly articulated through an experience or values‐based lens before identifying iterative questions. A qualitative inquiry may be best suited to answering these questions but is misaligned with the level 1 evidence that is sought to determine whether there is sufficient evidence in support of particular questions. Among the available level 1 evidence, methods for determining whether a question is answered or not can be subjective and available methodologies require greater scientific consideration as well as an understanding of the necessity for this in user‐defined priorities. In terms of including qualitative evidence, including metasyntheses may mitigate against the mismatch between the available evidence and user‐identified priorities somewhat as there is an increasing acceptance of these synthesis methods in healthcare decision‐making.^45^

## 6 CONCLUSION

Service‐user, carer and healthcare professional defined priorities focused on enhancing evidence for increasing community participation, ability to self‐manage and promoting recovery in daily life. Published research findings indicated that the priorities of service users, carers and healthcare professionals were aligned with researchers and funders in some areas and misaligned in others providing vital opportunities to develop research agendas that more closely reflect users' needs.

## AUTHOR CONTRIBUTIONS

Laoise Renwick drafted the manuscript. Laoise Renwick was responsible for the organization and preparation of the manuscript. Laoise Renwick was responsible for the concept of the priority‐setting exercise in collaboration with Rebecca Lauren Morris. Olivia Schaff devised the search strategy, and Olivia Schaff and Laoise Renwick at the Trust conducted the searches. Caitlin McWilliams and Susan Ramsdale assisted with project management. Laoise Renwick and Rebecca Lauren Morris were responsible to question development. Laoise Renwick had oversight of the steering committee, ranking and consensus workshop.

## CONFLICT OF INTEREST

The authors have no conflicts of interest to declare.

## Supporting information

Supporting information.

Supporting information.

## Data Availability

Data are available on request from the authors. The data that support the findings of this study are available from the corresponding author, upon reasonable request.
