# Supplementary material for: Stakeholder identified research priorities for early intervention in psychosis
Source: Health Expect. 2022 Sep 21;25(6):2960–70. doi: 10.1111/hex.13604 (PMC9700149; doi:10.1111/hex.13604)
Supplement: Supplementary file 1 — Supporting information. [file HEX-25--s001.pdf]

Vital Interventions for Psychosis Survey

Thinking about your own personal and/or professional experience with psychosis and early intervention teams, either delivering or receiving professional help, your experiences make your views important. We would like to hear your opinions about these services that are important to you. We are interested in learning about uncertain areas of practice or whether you think there are ways that early intervention services should help. To answer this question, it might be useful to ask yourself where you would focus resources for research if the decision were in your hands.

1 What questions do you have about how communities can empower or prevent you from being an active member of that community?

2 What questions do you have about recovery from psychosis?

3 What questions do you have about what we can we do for people who have ongoing symptoms or respond poorly to treatments that are offered?

4 What questions do you have about how NHS services, treatments, or interventions impact on your recovery?

5 What questions do you have about how to get help at the earliest opportunity?

6 What questions do you have about how services can help improve physical health?

7 Which of the following best describes you?
Person with psychosis
Partner/relative/friend of someone with psychosis
Nurse
Occupational Therapist
Psychiatrist
Psychologist
Support Worker
General Practitioner
Social Worker
Police Officer
Other Professional
Other

8 If you have experienced psychosis or are the relative or carer of someone that has, do you have any direct experience of early intervention in psychosis services?

9 If you are a healthcare professional, do you work in an early intervention in psychosis service?

10 How do you identify?
Female
Male
Prefer not to say

11 In what year were you born?

Thank you for taking the time to complete this survey.
