# Supplementary material for: Stakeholder identified research priorities for early intervention in psychosis
Source: Health Expect. 2022 Sep 21;25(6):2960–70. doi: 10.1111/hex.13604 (PMC9700149; doi:10.1111/hex.13604)
Supplement: Supplementary file 2 — Supporting information. [file HEX-25--s002.pdf]

Vital Interventions for Psychosis Survey

Early Intervention in Psychosis – how does it affect you and what services should do to help? Please write anything you think is important, ask a question, put a general topic area or something you've wondered about when you or someone you know with psychosis has attended services or been given a diagnosis.

1 Based on your involvement, what questions would you like researchers to answer about experiences of early psychosis and how to support people better?

2 Which of the following best describes you?
Person with psychosis
Partner/relative/friend of someone with psychosis
Nurse
Occupational Therapist
Psychiatrist
Psychologist
Support Worker
General Practitioner
Social Worker
Police Officer
Other Professional
Other

3 If you have experienced psychosis or are the relative or carer of someone that has, do you have any direct experience of early intervention in psychosis services?

4 If you are a healthcare professional, do you work in an early intervention in psychosis service?

5 How do you identify?
Female
Male
Prefer not to say

6 In what year were you born?

Thank you for taking the time to complete this survey.
